# Clinical service delivery implications of the COVID-19 pandemic on people with Inflammatory bowel disease: a qualitative study

**DOI:** 10.1186/s12913-023-10181-8

**Published:** 2023-11-02

**Authors:** Karen Kemp, Pearl Avery, Ruby Bryant, Amanda Cross, Kayleigh Danter, Andrew Kneebone, Deborah Morris, Amy Walker, Lisa Whitley, Lesley Dibley

**Affiliations:** 1 Manchester Royal Infirmary, Manchester, UK; 2 University of Manchester, Manchester, UK; 3 St Mark’s Hospital, Harrow, London, UK; 4 Patient and Public Involvement Group, Swansea, UK; 5 Gloucestershire Hospitals NHS Foundation Trust, Gloucester, UK; 6 Royal Bolton Hospital Foundation Trust, Bolton, UK; 7 East and North Hertfordshire NHS Trust, Stevenage, Hertfordshire UK; 8 South Warwickshire NHS Foundation Trust, Warwick, UK; 9 University College London Hospitals NHS Foundation Trust, London, UK; 10 Institute for Lifecourse Development, University of Greenwich, London, UK

**Keywords:** Inflammatory Bowel Disease, Clinical Services, Remote Access

## Abstract

**Background:**

During the COVID-19 pandemic, clinical services were severely disrupted, restricted, or withdrawn across the country. People living with Inflammatory Bowel Disease (IBD) – an auto-immune disorder for which medical treatment often results in immunosuppression, thus requiring regular monitoring—may have struggled to access clinical support. As part of a larger qualitative study, we investigated experiences of access to clinical services during the pandemic, and patient concerns about and preferences for services in the future.

**Methods:**

This exploratory qualitative study used semi-structured interviews to explore participants’ experiences of clinical services across the UK during the pandemic. All data were collected remotely (March – May 2021) using online video-calling platforms or by telephone. Audio files were transcribed professionally and anonymised for analysis. Data were analysed using thematic analysis.

**Results:**

Of the eight themes found across all data, four related specifically to accessing GP, local (district) hospital, and specialist (tertiary) referral services for IBD: 1) The Risk of Attending Hospital; 2) Missing Routine Monitoring or Treatment; 3) Accessing Care as Needed, and 4) Remote Access and The Future.

**Conclusions:**

Our findings support other studies reporting changes in use of health services, and concerns about future remote access methods. Maintenance of IBD services in some form is essential throughout crisis periods; newly diagnosed patients need additional support; future dependence on IBD services could be reduced through use of treatment / self-management plans. As the NHS digitalises it’s future services, the mode of appointment—remote (telephone, video call), or in-person – needs to be flexible and suit the patient.

## Background

When the Covid-19 pandemic reached the UK in late February/early March 2020, standard services across the NHS were disrupted, as all resources were redirected to support the massive increase in demand caused by overwhelmingly high numbers of hospital admissions and the acute care needs of critically ill patients with the SARS-CoV-2 virus. The structure of clinical services previously available to patients with chronic conditions, including those with Inflammatory Bowel Disease, changed rapidly. Inflammatory Bowel Disease (IBD) is an auto-immune condition comprised of a handful of specific diagnoses, including Crohn’s disease (CD), ulcerative colitis (UC) and IBD-Unclassified (IBD-U). These chronic illnesses have an unpredictable relapsing and remitting pattern, with symptoms of abdominal pain, fatigue, diarrhoea/constipation, and urgency, and are treatable but not curable. Careful and regular monitoring of medical management is essential to ensure timely intervention in the event of a relapse (or flare), to monitor the pharmacological and physiological impact of prescribed steroids, biologic, and biosimilar medications, and to detect the need for treatment review and/or referral for surgery. Routine screening includes blood monitoring for therapeutic levels, kidney and liver function, and blood count, alongside colonoscopies, Magnetic Resonance Imaging (MRI) screening and ultrasound procedures [1–3]. Regular prescriptions and blood tests were traditionally managed between hospital and GP services, but our previous work [4] has evidenced the impact of withdrawal of GP services on hospital-based IBD advice lines which, understaffed due to redeployment to COVID-19 services, struggled to cope with an often four-fold increase in call volume from patients seeking access to clinical support. The British Society of Gastroenterology (BSG) Risk Grid [5] provided IBD clinicians with an evidence-based means of assessing vulnerability to COVID-19 across their patient cohorts, to underpin clinical advice to shield, or to self-isolate as per Government guidelines.

## Methods

Using an exploratory qualitative approach, we aimed to understand the experiences of people with IBD who did or did not shield during the pandemic; this paper focuses on the data relevant to accessing clinical services. Participants were recruited from the patient charity Crohn’s & Colitis UK via their electronic newsletter, Facebook® page, or Twitter® feed, and were eligible to participate if they:

- Lived in any country of the United Kingdom (UK)
- Were at least 18 years old (no upper limit)
- Had a diagnosis of Crohn’s Disease, ulcerative colitis, or IBD-Unclassified confirmed before the onset of the pandemic in February 2020
- Had access to to a private phone for telephone interview, or were able to participate in video interview using Microsoft Teams®, on a device they already owned
- Were able to understand study procedures and give informed consent.

Patients who shielded were eligible if they met the preceding criteria AND were designated as extremely vulnerable to COVID-19 due to immunosuppression caused by medication for solid organ transplant or inflammatory conditions. There were no additional exclusion criteria.

**Fig. 1:**
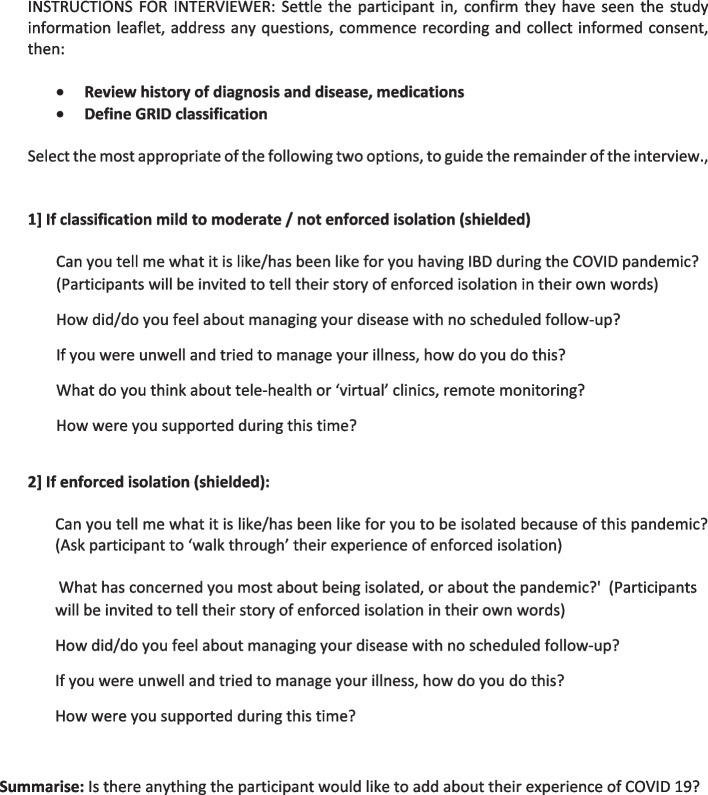
Interview schedule

Participants were assessed using the BSG GRID classification as either high, moderate, or low risk [5]. Following informed consent procedures, participants took part in either an online interview using video-calling software, or a telephone interview. Data were collected between March and May 2021, when the UK had emerged from the second lockdown and was embarking on the first of four ‘steps’ for a staged retraction of restrictions. The ‘stay at home’ order was still in place, and throughout the data collection period, a maximum of six people could gather together indoors, whilst up to 30 could meet outside [6]. The bespoke interview schedule [Fig. 1] was informed by content of calls to the IBD Advice Line at the first author’s workplace, which indicated high levels of concern regarding the pandemic amongst IBD patients. Interviews were recorded on a stand-alone Olympus digital audio recorder and transcribed professionally before being returned to the senior author (LD) for anonymisation prior to distribution for analysis.

### Method of data analysis

The qualitative data were analysed by the study team (one academic healthcare researcher, two Patient and Public Involvement [PPI] colleagues, and seven IBD Clinical Nurse Specialists). To ensure thoroughness, consistency and completeness, transcripts were distributed across the team according to capacity, and so that 10% of all transcripts were analysed at least three times [7]. Following the delivery of an online analysis training session by the senior author to the novice analysts, the guidance provided by Spencer et al. [8] was followed. Each analyst read through their allocated transcripts to get an overall sense of the data; they then worked through each transcript line by line, highlighting content of interest, and assigning an early label. Highlighted sections were then transferred into a data extraction table which recorded their initials, the ID label of the transcript, the page and line numbers of the extract, the extract itself, and the early label. These were then returned to the senior author, who collated all data tables to produce one master table [Table 1], from where extracts were sorted and organised into themes. This hands-on and immersive way of working with data embeds the researchers in the data and gives a complex insight into the experience being explored.

**Table 1:** Sample of first collation of extracted data into the master table

Interview number	Coder initials	Extract	Line numbers	Early theme/ideas
S-002	AW	Every infusion was really nerve-wracking. It’s better now, but to start with just coming into the hospital and being in the infusion unit – yes, that’s better now but to start with I was really anxious about that	184–186	Anxiety when attending hospital
S-016	KK	The difficulties were with getting my blood tests to monitor my liver, and things like that	35	Access/no access to healthcare
S-016	KK	Yes I remember the first time when I went into the GP to get the blood test probably about two or three months in [to the pandemic] I was looking around. It was very irrational basically and I knew it was fine, but it was just how many precautions can you take when you are just going to go and get a blood test?	144–147	The risk of attending hospital/GP for care
S-050	KK	It’s been difficult to be in touch with my actual consultant because I had an initial telephone consultation like just the regular one in July that was normal. I was then supposed to have one in January so a six-month check-up. I then kept on getting letters that that was then getting pushed back and back and back	175–178	Missing routine monitoring
S-011	DM	I don’t know why they’ve got rid of that phone line. I just don’t know if that was the right thing to do because when you are flared up it’s [essential]	92–93	Access/no access to healthcare
S-054	AW	Yes so I can always get a GP appointment within a few days. I’ve spoken to the IBD nurse as well, she phoned me back I think the next day so I’ve always had help there if I’ve needed it and been able to speak to someone quick enough	88–91	Access/no access to healthcare
S-029	DM	So in the past I’ve asked for phone appointments and couldn’t get them so I’m pleased that (virtual appointments) are there	172	Remote access and the future
S-011	DM	A lot of the time you do have to get [physically] checked over, they do have to feel and check	286	Physical/virtual attendance

During collation, themes were merged and refined further, and the resulting list of early themes and sub-themes was circulated to the team for comment and review. Following discussion via email with the team, and between the first and senior author, the final agreed themes were confirmed. Of the eight final themes, the four relating to clinical services are presented below. In keeping with qualitative methodologies, some explanation of meaning is provided alongside findings.

## Results

Of 44 participants recruited via the CCUK Twitter feed (*n* = 2), website (*n* = 12), e-newsletter (*n* = 13), Facebook page (*n* = 16) and chain referral from another participant (n = 1), 24 [55.8%] shielded; median age was 35 years [19–63 years]; 28 [63%] female; diagnosed with CD (*n* = 22), UC (*n* = 18), and IBD-U (*n* = 4). Median disease duration 10 years (0.9–36 years). Participants were assessed as high (*n* = 12), moderate (*n* = 23) and low (*n* = 9) risk against the BSG Risk Grid [5]; interviews lasted 40—94 min (mean 56 min, 36 s). 1 participant classified as Low and 1 as Moderate risk but with no guidance letter made their own decisions to shield due to other concerns/conditions; all high risks shielded, most low risks didn’t; of those at moderate risk, nine out of 23 chose not to shield. Shielding status changed over time as the pandemic progressed and/or vulnerability status changed (mostly due to medication changes, but sometimes due to mental wellbeing concerns). Table 2 reports each participant's status at the start of the pandemic.

**Table 2:** Risk level, shielding status and distribution of themes across participants

Participant ID, gender, age, diagnosis	BSG Risk status	Shielded		Theme 1: Risk of attending hospital	Theme 2: Missing routine monitoring or treatment	Theme 3: Accessing Care as Needed	Theme 4: Remote Access and The Future
	***Low***	***Yes***	***No***				
**S09 F, 54, CD**^**a**^	X	X			X	X	
**S13 F, 45, UC**	X		X		X		X
**S19 F, 54, UC**	X		X	X	X	X	
**S42 F, 25, UC**	X		X		X	X	X
**S46 F, 26, CD**	X		X	X	X	X	
**S54 M, 34, UC**	X		X		X	X	
**S62 F, 38, UC**	X		X			X	X
**S65 M, 30, CD**	X		X		X	X	
**S69 F, 35, UC**	X		X	X	X	X	
**N(%)**^**b**^	**9**	**1 (11.0)**	**8 (88.88)**	**3 (33.33)**	**8 (88.88)**	**8 (88.88)**	**3 (33.33)**
**(%)**^**c**^	**(20.45)**	**(2.27)**	**(18.18)**	**(6.81)**	**(18.18)**	**(18.18)**	**(6.81)**
	***Moderate***	***Yes***	***No***	**Theme 1: Risk of attending hospital**	**Theme 2: Missing routine monitoring or treatment**	**Theme 3: Accessing care as needed**	**Theme 4: Remote Access and The Future**
**S01 M, 54, UC**	X		X			X	X
**S02 F, 33,CD**	X	X		X		X	
**S03 M, 22, CD**	X		X		X		X
**S05 F, 42, CD**	X		X	X		X	X
**S07 F, 52, UC**	X	X			X	X	X
**S11 F, 43, CD**^**a**^	X	X		X	X	X	
**S16 F, 30, IBD-U**	X	X		X	X	X	
**S17 M, 31, UC**	X	X				X	X
**S18 M, 43, UC**	X		X	X	X		
**S21 M, 61, UC**	X	X		X			X
**S23 F, 32, CD**	X		X	X	X	X	
**S33 M, 22, UC**	X		X		X	X	
**S40 F, 28, UC**	X		X		X	X	
**S44 F, 54, UC**	X		X			X	X
**S45 F, 44, UC**	X	X			X	X	
**S48 M, 21, CD**	X		X			X	X
**S50 M, 30, UC**	X		X		X	X	X
**S53 F, 27, CD**	X	X		X		X	
**S56 F, 36, UC**	X	X		X		X	X
**S58 F, 40 IBD-U**	X		X			X	X
**S60 F, 62, UC**	X		X		X	X	X
**S61 M, 36, CD**	X	X				X	X
**S64 F, 22, IBD-U**	X	X				X	
**N(%)**^**b**^	**23**	**11 (47.82)**	**12 (52.17)**	**9 (39.1)**	**11 (47.82)**	**20 (86.95)**	**13 (56.52)**
**(%)**^**c**^	**(52.27)**	**(25.0)**	**(27.27)**	**(20.45)**	**(25.0)**	**(45.45)**	**(29.54)**
	***High***	***Yes***	***No***	**Theme 1: Risk of attending hospital**	**Theme 2: Missing routine monitoring or treatment**	**Theme 3: Accessing care as needed**	**Theme 4: Remote Access and The Future**
**S04 M, 63, CD**	X	X		X	X	X	X
**S06 M, 42, CD**	X	X		X	X	X	X
**S10 M, 35, CD**	X	X				X	X
**S15 F, 43, CD**	X	X				X	
**S24 F, 53, CD**	X	X		X	X	X	
**S26 F, 55, IBD-U**	X	X		X	X	X	X
**S28 F, 35, CD**	X	X		X		X	
**S29 M, 19, CD**	X	X		X	X	X	
**S31 F, 22, CD**	X	X				X	X
**S32 F, 27, CD**	X	X		X		X	
**S37 F, 31, CD**	X	X		X		X	X
**S71 M, 32, CD**	X	X		X	X	X	
**N(%)**^**b**^	**12**	**12 (100)**	**0**	**9 (75.0)**	**6 (50.0)**	**12 (100)**	**6 (50.0)**
**(%)**^c^	**(27.27)**	**27.27)**		**(20.45)**	**(13.63)**	**(27.27)**	**(13.63)**
**TOTAL N (%)**^**b**^	**44**	**24 (54.54)**	**20 (45.45)**	**21 (47.72)**	**25 (56.81)**	**40 (90.90)**	**22 (50.0)**

*BSG* British Society of Gastroenterology, *CD* Crohn’s disease, *IBD-U* IBD-unclassified, *UC* Ulcerative colitis

^a^Opted to shield in absence of guidance letter due to self-perceived levels of enhanced risk

^b^As number and percentage of participants per category

^c^As percentage of all participants

Four clinically focussed themes emerged: *The Risk of Attending Hospital*, *Missing Routine Monitoring or Treatment*, *Accessing Care as Needed* and *Remote Access and The Future*. Themes emerged across the data and have also been categorised according to risk and shielding status [Table 2]. Data extracts presented below are labelled with Study ID number, gender, diagnosis, age, risk level (Hi/Mod/Lo), shielding status (FS = Fully Shielded; PS = Partially Shielded; NS = Not Shielded).

### Theme 1: The risk of attending hospital

Confronted by daily media reports on the severity of the national and global pandemic situation, and advised to either stay at home if shielding, or go out only for essential purposes such as food shopping if isolating, participants felt that their essential hospital visits were high risk. Some were concerned about perceived poor hygiene practices by healthcare staff:

When you go and see (the Consultant) his mask is not even on, it’s down his face, you *can see his nose. He touches it half a dozen times and that makes me anxious. *(S-29,M,CD,19,Hi,FS).

Others reported varying levels of concern linked to hospital visits, starting from a point of raised anxiety about attending for necessary infusions which gradually reduced over time, to finding visits much less stressful than anticipated:

Surprisingly, going into hospital wasn't as stressful as I thought it would be. I went for my infusions and I think because [the hospital] was quite empty and I knew I had to do it, there wasn't much point stressing about it. Everyone was wearing masks andyou had lots of space—usually the hospital is teeming with people and it was really Empty, so it felt okay. (S-32,F,CD,27,Hi,FS).

The expected and actual anxieties about the risk of attending clinical areas during the pandemic, have been reflected in other literature reporting patients who have avoided seeking necessary care due to similar concerns [9, 10]. Participants who did not actually need to attend, worried about the perceived risk of potential visits.

### Theme 2: Missing routine monitoring or treatment

Participants felt unsettled when the usual pattern of care they were used to was suddenly disrupted. Some reported that gastroenterology appointments ‘just disappeared’ whilst others, aware of the necessity of keeping on top of their symptoms through regular monitoring, were concerned that something could get missed:

I’m not having [tests] done—what is going to happen if there was something they’d have picked up and it’s not being done? Is there something going on that I don’t know About? (S-09,F,CD,54,Lo,FS).

There is wide variation in the way that IBD ‘behaves’ and close and careful monitoring is often essential to detect early signs of potential problems. Participants worried about the consequences of missing routine monitoring and treatment:

What you realise is that if you are missing your appointments or your doses of medication that’s when it hits a little bit closer to home that hey, my health is not as good and I actually need these services as often as I used to get it pre-COVID but COVID took some of them away. (S-28,F,CD,35,Hi,FS).

For some, their determination to ensure monitoring continued, led them to be extremely pro-active in securing appointments:

I knew I needed my monitoring and I’m really keen to have the calprotectin done regularly. So I just had to be sharp elbowed in that way and push for an appointment. (S-19,F,UC,54,Lo,FS).

These words portray a powerful image of determination to ensure that personal healthcare needs were met, despite the very challenging landscape during the pandemic. This participant had been living with UC for 35 years – others with less experience of IBD and perhaps also less confidence may not have been able to be so self-determining.

One participant reported funding essential monitoring privately, realising their responsibility for keeping themselves well:

I’ve actually been having my bloods done privately … I’ve been paying and having a full blood count, CRP … I thought I’m really going to take this onboard a little bit and have a look myself and try and monitor all my levels and just make sure that I can help myself as best as I can throughout this, again probably to help not flare. (S-002,F,CD,33,Mod,FS).

This approach reflects what was seen elsewhere in the data – in varying ways, people with IBD started to take on a level of responsibility for their IBD that they had not accepted before the pandemic.

### Theme 3: accessing care as needed

Where the previous theme focussed on routine monitoring and treatment, this theme addresses concerns for those who needed more urgent support and could not always access it. Some reported closure of the telephone advice line, and seemingly did not know how to now contact their IBD team. Others reported lengthy delays in securing appointments:

I was told that I’d have to wait until January for me to even just get an initial Appointment which, the way I was feeling, it was too long (S-2,F,UC,25,Lo,NS) or in following up issues of concern: It’s taken from October to December for me to have an MRI scan and then it’s taken until 31st March for them to call and say ‘This is what we’ve seen’. Now my appointment is on 26th May for them to say this is what we’re going to do, and that’s the first time they’re inviting me into clinic since it happened. It’s very frustrating. (S-53,F,CD,27,Hi,FS).

As in many chronic conditions, effective management of IBD depends on early intervention to prevent escalation of the problem; considering this, the concern these participants expressed about these delays are understandable.

Occasionally, participants reported relatively easy and rapid access to their GP and the IBD nurses, commenting that ‘*I’ve always had help there if I’ve needed it and been able to speak to someone quick enough’* (S-54,M,UC,34,Lo,NS).

These experiences highlight the variation in service access and delivery across the UK, likely to have been influenced by the size of the multidisciplinary IBD team, the extent of redeployment of team members to frontline COVID duties, and the availability of space and resources to restructure and be able to safely maintain essential services [11].

### Theme 4: Remote Access and The Future

The pandemic saw a rapid escalation of remote access options, with virtual (telephone or online) appointments replacing in-person appointments in outpatient settings. Amongst our participants, opinion was divided regarding the prospect of remote or virtual appointments continuing post-pandemic. Some were happy with any mode of contact:

It’s nice to have a face to face [appointment] every six months but more than happy Just to have a phone call or an email just to check in (S-17,M,UC,31,Mod,PS).

Others, who did not trust themselves to spot early signs of a change in their clinical status, were not keen to embrace remote access options:

There’s been talk of having [remote video] appointments in the future and I really don’t like that idea because I want my gastroenterologist to touch my tummy and see what it’s like—I don’t want to be in a system where it’s basically relying on me giving. My symptoms online. (S-05,F,CD,42,Lo,NS).

This diversity in opinion perhaps reflects the different requirements of those with stable disease, and those with more changeable symptoms:

If someone is having a flare you need to be physically seen by someone. It’s no good having it virtually because they can’t inspect you, can’t feel for anything in your stomach, blood tests, all those things. Whereas if you are not having a flare and you are – not right as rain but you don’t have symptoms – then yes, I’m all for virtual, but I Think it depends on where you are clinically (S-42,F,UC,25,Lo,NS).

These patient perspectives strongly suggest that the mode of future appointments need to be guided by the clinical status of the individual at the time, and perhaps their level of experience and confidence with their IBD.

## Discussion

During the pandemic, participants who were low risk and did not need to shield were mostly concerned about missing their routine monitoring and treatment and being able to access care if they needed it. Participants assessed as being at moderate risk mostly made their own decisions, based on other factors in their lives, on whether and how much to shield. Just under one third of this group worried about the risk of attending hospital during the pandemic, whilst the majority (*n* = 20, 86.95%) were mostly concerned about accessing care as needed, with a range of experiences from no access at all, to what was perceived as normal service, being described. Approximately 50% of this group reported concerns about missing routine monitoring or treatment and commented on the likelihood of remote consultation options in the future. All participants who were identified as high risk, shielded fully. It is notable that in this group of 12 participants, all but one had a confirmed diagnosis of CD. They also had a higher rate of concern about the risks of attending hospital (75% versus 39.1% for moderate risk, and 33% for low risk), likely explained by their classification as high risk being an indicator of the severity of their illness and therefore a greater possibility that they may need hospital care. All members of the high-risk group reported being able to access care when they needed it, during the pandemic. It is possible that the provision of ‘hot’ or ‘flare clinic’ was only communicated to those seeking urgent care, leaving others to worry about clinical support, unaware of this initiative. Better communication about the support structures that were in place, may have reduced anxiety amongst those in all risk groups, who perhaps would have felt reassured that help would have been available, had it been needed. We did not find that particular themes were more or less relevant to participants by age, gender, or diagnosis, and it was not the aim of this qualitative study to determine such correlations. A larger cross-sectional survey, based on the findings of this study, may help to clarify any potential associations.

The findings reported here add to a growing body of evidence detailing the experiences of patients relating to accessing healthcare during the first and second waves of the pandemic. Such experiences are necessarily informed and influenced by the context in which these occurred, and it is likely that news and social media reports, as well as past experience of their condition, informed participants perceptions. For example, anyone recently diagnosed or who had endured a lengthy struggle to find effective treatment and get their IBD under control, would respond differently to the perceived threats the pandemic presented in terms of continuity of care, than someone with mild, or very well-controlled disease, or lengthy self-management experience.

The disruption to routine services had a specific impact on those living with chronic conditions including IBD, for whom regular monitoring and treatment is essential to maintain optimum health [12] and continuity with the same clinical team is important to avoid lack discontinuity of care [13]. Whilst there may have been beneficial reductions in footfall and demand on services – for example, a drop in non-urgent visits to Accident and Emergency (A&E) departments – there were also worrying reductions in attendance and admissions globally for critical and urgent conditions [14–16] and amongst paediatric patients [17]. In Greece and the UK and perhaps reflected elsewhere in the world, striking reductions were noticed in the numbers of people seeking help for cardiovascular symptoms including cardiac events and stroke [15, 18]. In the UK, attendances at A&E departments fell by 25% in the week following the first lockdown in March 2020 and whilst a proportion of these may have been non-urgent visits, it raises concerns that those who needed to, either would not or could not access necessary services [19]. Disruption to services, and to routine care and monitoring, is likely to have a long-lasting impact on health-related outcomes for those with chronic conditions [20] with ‘delayed diagnoses, procedures and surgeries’ in gastroenterology ‘undoubtedly resulting in increased morbidity and mortality’ [21]. Early intervention is essential to ensure the best outcomes for patients with IBD [22] and delays to follow-up such as those expressed in our data, are likely to have negative consequences for some patients [23].

Whilst one of the aims in chronic conditions is to promote self-management and self-efficacy, unprecedented events such as the COVID-19 pandemic disrupted the ability of some patients to be self-determining in managing their IBD, causing some frustration. Conversely, the inability to rely on the NHS prompted others to take on a greater level of responsibility for the management of their IBD, suggesting that enhancing self-management capabilities could reduce pressures on very stretched services by reducing demand. However, as Moran et al. have highlighted [24] self-care needs to be supported during uncertain times. Our data confirms that services need to be maintained in some form, during crisis periods, with changes to access communicated clearly to patients.

Some of our findings directly contradict those reported previously by Harris and colleagues [25] who conducted an online survey of IBD patients categorised as either high or moderate risk, as per the BSG Grid [5] used above. Specifically, where Harris et al.[25] reported that ‘most services were largely uninterrupted’ and that ‘patients expressed a strong wish of having future care delivered remotely, even during IBD flares’ (p.1) our findings resonated with the alternatives reported by Kennedy et al., [11]; for many of our participants, access to services was significantly disrupted and there were very mixed feelings about the mode of future care delivery, with a strong message that this needed to suit the individual at the time. Harris et al. do point towards a need for flexibility, with patients indicating a preference for different modes of appointments according to their clinical status. However, whereas they report that remote access modes were less acceptable to those over 55 years of age in either relapse or remission, our findings suggest that there is also concern about remote access in younger patients. In the UK, the pandemic has prompted an expectation of reducing physical outpatients’ appointments by 25% by 2024 [26, 27]. Remote and flexible access options are a necessary part of the future healthcare landscape, but those with chronic conditions, including IBD, will need to be able to access care as required, using strategies such as patient-initiated follow-up [28, 29].

### Limitations

At the time of data collection (March – May 2021, just as the second lockdown ended, face to face data collection was impossible due to continuing restrictions. Remote methods were necessary however it is possible that digital poverty, precluding access via telephone or video platform, may have prevented participation for some. As with all studies, a recruitment bias exists when people choose to take part or not. It is also possible that our recruitment methods and source may have attracted a particular demographic range, and those with access to digital platforms. However, the heterogenous sample described above demonstrates a varied participant profile.

## Conclusions

The COVID-19 pandemic presented people living with IBD with a range of challenges relating to accessing health care – from concerns about the pandemic-related risks of entering the clinical environment to receive routine therapeutic interventions, to losing necessary routine and follow-up appointments, securing urgent support, knowing how to access changed services, and concerns about future remote models of care. Apart from the requirement to ensure that changes in care provision are adequately communicated to all patients with IBD, there is no distinct pattern of responses that would enable clinicians to expect certain sub-groups of patients with IBD to require a particular approach to care, and the wisest approach is to assess need on an individual basis.

Our findings add to the growing body of qualitative and quantitative evidence that details patients’ varied experiences during lockdown – experiences which should influence the delivery of clinical services and planning for major disruptive events in the future.

## Data Availability

The datasets generated and analysed during the current study are not publicly available because study participants gave permission for anonymised data supporting this study to be used in future studies only by members of this study team. The data has therefore not been placed in a recognised repository but is available from the corresponding author on reasonable request to anyone delivering research studies with any member of this study team, at any point in the future.

## Abbreviations

A & E: Accident and Emergency
BSG: British Society of Gastroenterology
CD: Crohn’s Disease
IBD: Inflammatory Bowel Disease
IBD-U: IBD-Unclassified/Undifferentiated
NHS: National Health Service
UC: Ulcerative Colitis
